# S100A8/A9-MCAM signaling promotes gastric cancer cell progression via ERK-c-Jun activation

**DOI:** 10.1007/s11626-025-01105-3

**Published:** 2025-09-09

**Authors:** Youyi Chen, Xu Yang, Rie Kinoshita, Nahoko Tomonobu, Bo Pan, Fangping Wu, Xu Zhang, Kazumi Sagayama, Bei Sun, Masakiyo Sakaguchi

**Affiliations:** 1 Department of Breast Surgery, The First Affiliated Hospital, Zhejiang University School of Medicine, Hangzhou, Zhejiang Province China; 2 Department of Pathology, The First Affiliated Hospital, Zhejiang University School of Medicine, Hangzhou, Zhejiang Province China; 3 Department of Cell Biology, Okayama University Graduate School of Medicine, Dentistry, and Pharmaceutical Sciences, 2-5-1 Shikata-cho, Kita-ku, Okayama-shi, Okayama, 700-8558 Japan; 4 Department of Biochemistry, Kawasaki Medical School, Kurashiki, Okayama, Japan; 5 The First Affiliated Hospital, Zhejiang University School of Medicine, 79 Qingchun Road, Hangzhou, 310003 China; 6 School of Pharmaceutical Sciences, Zhejiang Chinese Medical University, Hangzhou, 310000 China; 7 Department of Urology, The First Affiliated Hospital, Zhejiang University School of Medicine, 79 Qingchun Road, Hangzhou, 310003 China; 8 Faculties of Educational and Research Management Field, Okayama University, Okayama, Japan; 9 Department of Pancreatic and Biliary Surgery, The First Affiliated Hospital of Harbin Medical University, Harbin, 150001 China

**Keywords:** Gastric cancer, S100 protein, MCAM, Inflammation, Metastasis

## Abstract

S100 protein family members S100A8 and S100A9 function primarily as a heterodimer complex (S100A8/A9) in vivo. This complex has been implicated in various cancers, including gastric cancer (GC). Recent studies suggest that these proteins play significant roles in tumor progression, inflammation, and metastasis. However, the exact mechanisms by which S100A8/A9 contributes to GC pathogenesis remain unclear. This study investigates the role of S100A8/A9 and its receptor in GC. Immunohistochemical analysis was performed on GC tissue samples to assess the expression of the S100A8/A9 receptor melanoma cell adhesion molecule (MCAM). In vitro transwell migration and invasion assays were used to evaluate the motility and invasiveness of GC cells. Cell proliferation was assessed using a growth assay, and Western blotting (WB) was employed to examine downstream signaling pathways, including ERK and the transcription factor c-Jun, in response to S100A8/A9–MCAM interaction. S100A8/A9 stimulation enhanced both proliferation and migration through MCAM binding in GC cell lines. These cellular events were accompanied by ERK activation and c-Jun induction. Downregulation of MCAM suppressed both ERK phosphorylation and c-Jun expression, highlighting the importance of the S100A8/A9‒MCAM‒ERK‒c-Jun axis in promoting GC progression. These findings indicate that S100A8/A9 contributes to GC progression via MCAM, which activates the ERK‒c-Jun pathway. The S100A8/A9‒signaling axis may represent a novel therapeutic target in GC.

## Introduction

Gastric cancer (GC) remains one of the most prevalent and deadly malignancies worldwide. The prognosis for patients with advanced or metastatic GC remains poor, with 5-year survival rates typically falling below 30%. A major challenge in GC research lies in its substantial molecular and histological variability. This complexity hinders the identification of universally effective therapeutic targets and complicates efforts to predict clinical outcomes. Furthermore, GC is strongly shaped by the tumor microenvironment, especially by chronic inflammation and immune modulation, both of which contribute to tumor invasion, metastasis, and resistance to conventional therapy (Tan* et al.* 2015; Ajani* et al.* 2016). Thus, uncovering the molecular mediators that connect inflammation to tumor progression is critical for improving GC diagnosis and treatment.

The S100 family of calcium-binding proteins has emerged as a central class of inflammation-associated mediators in cancer. In particular, S100A8 and S100A9—two highly homologous proteins that typically dimerize to form calprotectin—regulate multiple processes relevant to tumor biology, including immune response, epithelial–mesenchymal transition (EMT), angiogenesis, and metastasis. We previously identified two key receptors involved in melanoma organ-specific metastasis: extracellular matrix metalloproteinase inducer (EMMPRIN) and melanoma cell adhesion molecule (MCAM) (Hibino *et al.* 2013; Ruma* et al.* 2016; Sakaguchi* et al.* 2016). Notably, our findings in a mouse model demonstrate that EMMPRIN interacts preferentially with S100A9, and this interaction promotes melanoma cell entry into the bloodstream and accumulation in skin tissue artificially enriched with S100A9 (Hibino *et al.* 2013). MCAM, which responds to S100A8/A9, promotes lung-specific metastasis in melanoma cells expressing this receptor (Ruma* et al.* 2016). These receptors may function cooperatively in melanoma cells to facilitate S100A8/A9-driven organ-specific metastasis. Previous studies have shown that EMMPRIN initiates a downstream signaling cascade culminating in NF-κB activation via the TRAF2 adaptor protein (Sakaguchi *et al*. 2016). However, significant gaps remain in elucidating the downstream signaling pathways of MCAM in different cancer types, particularly melanoma and breast cancer (Wang* et al.* 2020; Mannion* et al.* 2023; Balcioglu* et al.* 2024; Braun* et al.* 2025).

Building upon these findings, our team has also explored the regulatory networks controlling S100A8/A9 expression in tumor cells. In our prior investigations, we demonstrated that ETS translocation variant 4 (ETV4), also referred to as polyoma enhancer activator 3 (PEA3), located downstream of MCAM, serves as a critical transcription factor (TF) that drives melanoma metastasis through robust upregulation of matrix metalloproteinase-25 (MMP25). Additionally, we identified mitogen-activated protein kinase kinase kinase 8 (MAP3K8, also known as TPL2), which interacts with the cytoplasmic tail of MCAM, plays a pivotal role in the activation of ETV4 (Chen* et al.* 2019a). We also reported a crucial role of MCAM in breast cancer in response to extracellular S100A8/A9, underscoring the indispensable function of inflammatory cytokines in cancer-associated microenvironments. Downstream signaling analyses showed that MCAM drives the activation of ETV4, which in turn modulates the expression and activity of ZEB1 (Chen* et al.* 2019b) but not MMP25 observed in melanoma (Chen* et al.* 2019a).

Given the distinct pro-tumorigenic role of MCAM in cancer cells, as described above, our attention turned to its role in GC, where MCAM expression is also highly upregulated. In the present study, we aimed to investigate whether S100A8/A9 participates in GC progression, similar to its role in melanoma and breast cancers, via MCAM. Clarifying the downstream signaling cascades driven by S100A8/A9–MCAM interaction may improve our molecular understanding of GC and support future diagnostic and therapeutic advances.

## Materials and methods

### Cells and reagents

All gastric cell lines (GES-1, AGS, HGC-27, KATO III, NCL-N87, and MGC-803) were maintained in RPMI 1640 medium (Corning Inc., Tewksbury, MA) supplemented with 10% fetal bovine serum (FBS) (Thermo Fisher Scientific, Waltham, MA) and 1% penicillin–streptomycin at 37 °C in a humidified incubator with 5% CO_2_. Cells were dissociated using 0.25% trypsin and 0.02% EDTA for an appropriate duration.

All cell lines were confirmed to be mycoplasma-negative by Hoechst 33,342 staining under both live and fixed conditions. In addition, all cell lines were authenticated by DNA profiling using short tandem repeat (STR) analysis for 21 representative human loci, confirming identity with GES-1, AGS, HGC-27, KATO III, NCL-N87, and MGC-803 cells. There was no evidence of intercellular contamination, thereby ensuring the purity of the cell lines. Cell line authentication was performed by Shanghai Biowing Biotechnology Co. Ltd. (Shanghai, China) for GES-1, AGS, HGC-27, NCL-N87, and MGC-803, and by Procell Life Science & Technology Co. Ltd. (Wuhan, China) for KATO III.

### Preparation of recombinant protein and antibody

Highly purified human recombinant S100A8/A9 heterodimer protein was prepared as described (Futami* et al.* 2016). A mouse monoclonal anti-S100A8/A9 neutralizing antibody (designated Ab45) was developed in-house as reported (Kinoshita* et al.* 2019; Araki *et al.* 2021; Gohara* et al.* 2025).

### Plasmids

The mammalian gene expression constructs used in this study were generated using the pIDT-SMART-C-TSC vector (pCMViR-TSC) as a backbone to achieve high-level expression of the inserted cargo genes (Sakaguchi* et al.* 2014, 2024). The cDNAs inserted into the multi-cloning site of pCMViR-TSC were designed to be expressed as C-terminal 3xHA‒6His-tagged fusion proteins. The cDNAs encoding GFP, wild-type MCAM (MCAM WT), and a dominant-negative MCAM lacking the C-terminal cytoplasmic tail (MCAM DN) (Ruma* et al.* 2016) were inserted into the multi-cloning site of pCMViR-TSC. Transient transfection of these plasmids into cultured cells was performed using jetPRIME (Polyplus Transfection, Inc., Illkirch, France) following the manufacturer’s instructions.

### siRNA

Human MCAM siRNA (#1, ID No. s8571; #2, ID No. s8572) and control siRNA (Silencer Negative Control siRNA #1) were purchased from Thermo Fisher Scientific. The siRNAs were transfected using Lipofectamine RNAiMAX reagent (Thermo Fisher Scientific).

### RNA-Seq

Total RNA was isolated using TRIzol reagent (Thermo Fisher Scientific) following the manufacturer’s instructions. RNA purity and concentration were assessed using a NanoDrop spectrophotometer (Thermo Fisher Scientific), and RNA integrity was verified by agarose gel electrophoresis. RNA-Seq was performed by Oebiotech (Shanghai, China) using the Illumina NovaSeq platform with 2 × 150 bp paired-end reads. Differential gene expression analysis was conducted using R (ver. 4.1.0) with appropriate packages. Enrichment analysis was performed on the upregulated genes with a false discovery rate of less than 0.05 to determine which cellular responses these gene upregulations evoked.

### Western blot (WB)

Proteins were extracted from the cells using RIPA lysis buffer (Beyotime Ltd., Shanghai, China) and diluted to 2 mg/ml in 4X SDS loading buffer. After heat denaturation, equal amounts of protein were resolved on a 4–20% SDS‒polyacrylamide gel electrophoresis (SDS‒PAGE) gel and transferred to a PVDF membrane. The membrane was blocked with 5% skim milk at room temperature for 1 h, cut as needed, and incubated overnight at 4 °C with primary antibodies. The following day, after three washes with TBST, the PVDF membrane was incubated with horseradish peroxidase (HRP)-conjugated secondary antibody at room temperature for 1 h. Protein bands were visualized using enhanced chemiluminescence detection reagent. All primary antibodies were purchased from Cell Signaling Technology and included rabbit anti-MCAM (MCAM, E3F3E) antibody, rabbit anti-human phospho-p44/42 MAPK (Erk1/2) (Thr202/Tyr204) antibody, rabbit anti-human p44/42 MAPK (Erk1/2) (137f5) antibody, rabbit anti-human phospho-SAPK/JNK (Thr183/Tyr185) (81E11) antibody, rabbit anti-human SAPK/JNK antibody, rabbit anti-human phospho-p38 MAPK (Thr180/Tyr182) (D3F9) antibody, rabbit anti-human p38 MAPK (D13E1) antibody, rabbit anti-human c-Jun (60A8) antibody, rabbit anti-beta-actin (13E5) antibody, and anti-rabbit IgG, HRP-linked antibody.

### MTT assay

Cell proliferation was assessed using the Celltiter 96® Aqueous One Solution Cell Proliferation assay (MTS; Promega BioSciences, San Luis Obispo, CA). Cells were trypsinized, counted, and seeded into 96-well plates with six replicate wells per group. After adherence, the medium was replaced with a 1:10 mixture of MTS reagent and culture medium. A total of 110 µl of this mixture was added to each well and incubated for 2 h at 37 °C in a humidified incubator with 5% CO_2_. Absorbance (optical density) was measured using a microplate spectrophotometer.

### Colony formation assay

After the cells were trypsinized and counted, they were suspended in medium to a density of 1500 cells/ml and seeded in six-well plates (3000 cells per well) in triplicate per group. Cells were cultured for 14 d in a humidified incubator or until most colonies reached > 50 cells, as confirmed microscopically. Medium was replaced every 3 d, and colony growth was monitored throughout. After colony formation, cells were fixed with 4% formaldehyde and stained with crystal violet.

### Transwell assay

Transwell assays were performed to evaluate cell invasion and migration capacities. Cells in the logarithmic growth phase were trypsinized, counted, and resuspended at 1 × 10^5^ cells/ml. Transwell inserts were placed into 24-well plates containing 600 µl of 10% FBS medium with or without S100A8/A9 antibody in the lower chamber, and 200 µl of 0.5% FBS medium with 2 × 10^4^ cells was added to the upper chamber. After 24–48 h, cells were fixed with formaldehyde, stained with crystal violet, and imaged under a light microscope.

### Study samples and grouping of samples

The clinical GC tissue specimens, collected with the ethical approval numbers 846 and 1155, were used in this study, which was authorized by the Clinical Research Ethics Committee of the First Affiliated Hospital, College of Medicine, Zhejiang University, ensuring the study’s integrity and reliability. A total of 54 formalin-fixed and paraffin-embedded tissue (FFPE) samples from GC patients were retrospectively collected from the Pathological Sample Bank of the Department of Pathology, the First Affiliated Hospital of Zhejiang University School of Medicine. All these patients were pathologically confirmed to have GC. The collected samples were grouped according to the degree of differentiation and pathological TNM staging. The degree of differentiation was evaluated by two highly experienced pathologists, whose expertise and knowledge in the field ensured the accuracy of the evaluation. They based their evaluation on the histological characteristics of the tumor cells, and the relevant international standards and guidelines for GC staging were used to determine the pathological TNM staging.

### Immunocytochemistry and immunohistochemistry

The tissue specimens were fixed with 4% paraformaldehyde in phosphate-buffered saline (PBS) and then treated with ethanol. After deparaffinization, the tissue samples were pretreated by microwave heating. At the same time, the cultured cells were fixed using a method similar to that described above and prepared for immunocytochemistry. The fixed tissue sections and cultured cells were incubated with the primary antibody at 37 °C for 1 h, followed by the application of a second antibody, either Alexa594-conjugated goat anti-mouse IgG antibody (Thermo Fisher Scientific) or Alexa488-conjugated goat anti-rabbit IgG antibody (Thermo Fisher Scientific). The primary antibody used was a rabbit anti-MCAM (E3F3E; Cell Signaling Technology).

### Statistical analysis

Each experiment was repeated three times, and the resulting raw data were statistically analyzed. The calculated values are mean ± standard deviation (SD). One-way analysis of variance (ANOVA) was performed for the comparative evaluation of more than two groups. When the ANOVA showed a significant difference, the Bonferroni post hoc test was used. Probability (*p*) values < 0.05 were considered to indicate statistical significance.

## Results

### MCAM is selectively expressed in poorly differentiated GC tissues

**Figure 1.**
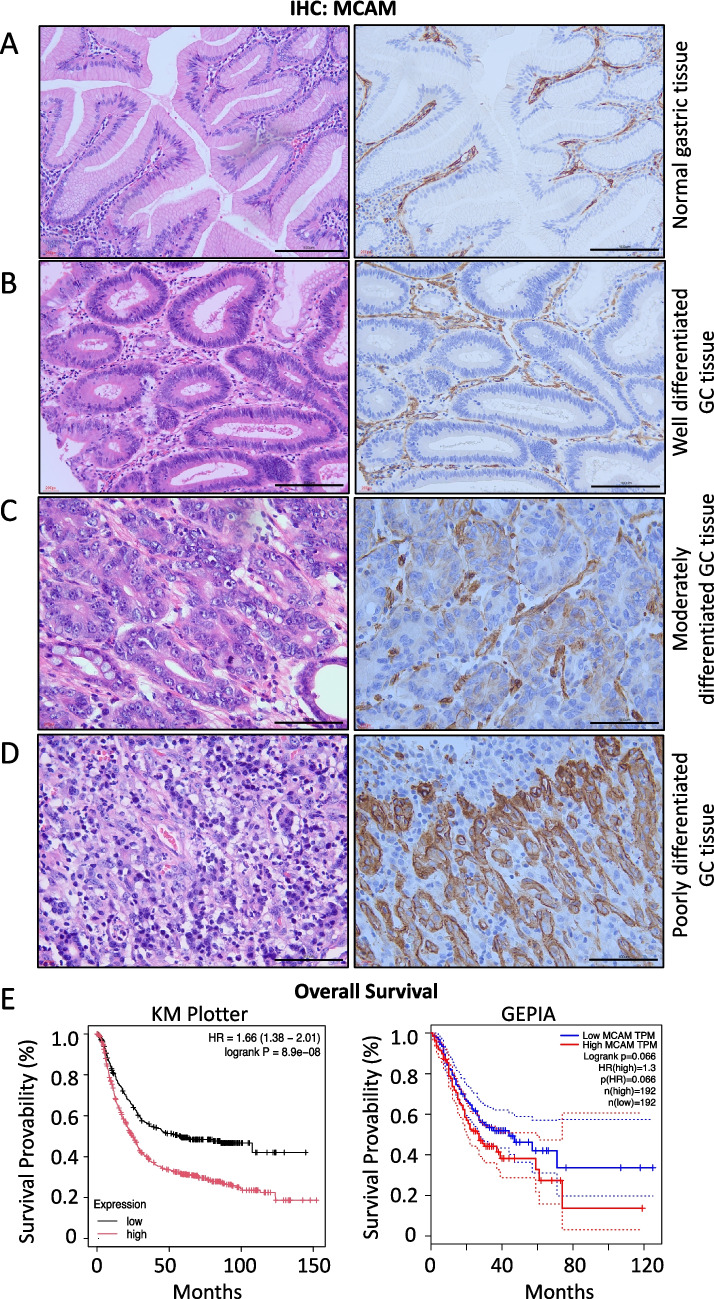
Immunohistochemical analysis of MCAM in GCs. (A-D): Immunohistochemistry. Normal gastric mucosa tissue (*A*) and GC tissues at different grades (well- differentiated type (*B*), moderately differentiated type (*C*), and poorly differentiated type (*D*), the most advanced grade) were prepared clinically. Samples were stained with hematoxylin and eosin (H&E) to examine histology (*left*) and with an anti-MCAM antibody to assess MCAM distribution and expression intensity (*right*). *Scale bars*: 100 μm. (*E*) Online survival analysis of GC patients based on MCAM expression using publicly available databases (Kaplan-Meier Plotter: https://kmplot.com/analysis/ (left), and GEPIA: http://gepia.cancer-pku.cn/ (right)).

To investigate the relationship between MCAM expression and histological differentiation in human GC tissues, we performed immunohistochemical (IHC) staining on a series of gastric tissue samples ranging from normal gastric epithelium to poorly differentiated adenocarcinomas. The MCAM expression levels were evaluated and correlated with the degree of pathological differentiation. In normal gastric mucosal epithelium (Fig. 1*A*), no MCAM immunoreactivity was observed in either surface epithelial cells or glandular structures, aside from the interstitial stromal areas. Similarly, MCAM expression was limited to non-cancerous stromal region cells. It was undetectable in well-differentiated gastric adenocarcinoma cells, which formed a clearly distinguishable epithelial layer separated from the stroma (Fig. 1*B*). These well-differentiated cancers, which retain some structural and functional characteristics of normal gastric epithelium, showed no detectable MCAM staining, suggesting that MCAM is not involved in the early or less aggressive stages of GC progression.

On the other hand, we detected a low but discernible MCAM expression within moderately differentiated gastric adenocarcinoma epithelial cells (Fig. 1*C*). The IHC analysis showed distinct membranous and partial cytoplasmic staining in tumor epithelial cells, suggesting an upregulation of MCAM during the intermediate stages of cancer progression. The intermediate expression level may reflect a transition state in which cancer cells acquire more invasive and aggressive characteristics while retaining some degree of differentiation. Notably, MCAM expression was markedly elevated in poorly differentiated GC tissues (Fig. 1*D*). These samples displayed strong and widespread MCAM staining in the membrane and cytoplasm of cancer cells. This intense expression was consistently present in multiple poorly differentiated specimens, indicating a robust upregulation of MCAM in more aggressive cancer phenotypes. The elevated level of MCAM in these samples supports the hypothesis that MCAM contributes to malignant phenotypes, including enhanced motility, invasiveness, and potential resistance to therapy. This finding is further supported by in silico survival analysis of GC patients using publicly available datasets, which revealed a strong association between elevated MCAM expression and poor survival outcomes, consistently observed across two platforms, KM plotter and GEPIA (Fig. 1*E*).

### Correlation between MCAM expression and clinicopathological characteristics

As shown in Table 1, we explored the connections between MCAM expression and various clinicopathological characteristics in GC tissues. It is important to note that our study was unable to assess MCAM expression in M-stage specimens due to the lack of graded specimens in our stratified collection. However, our research has successfully demonstrated a significant association between MCAM expression and histological alteration, T stage, and N stage in GC. These findings offer valuable insights into the potential role of MCAM in the invasive and metastatic progression of GC. Specifically, MCAM expression levels increased as differentiation declined, indicating higher expression in poorly differentiated tumors than in moderately or well-differentiated ones (*p* = 0.001). Moreover, elevated MCAM expression increased with advancing T stage (tumor invasion depth), with significantly higher levels observed in T3–T4 tumors compared to T1–T2 (*p* = 0.001). Similarly, MCAM expression was significantly associated with lymph node involvement (N stage), with higher MCAM levels detected in N2–N3 cases than in N0–N1 (*p* = 0.007). These results suggest that MCAM expression is closely related to tumor invasive aggressiveness and metastatic progression, and importantly, they underscore the potential of MCAM as a biomarker for advanced GC, with possible diagnostic utility.

**Table 1.** Correlation between MCAM expression and clinicopathological characteristics of GC tissues

Feature	Category	*N*	MCAM negative (*n* = 37)	(*n* %)	MCAM positive (*n* = 17)	(*n* %)	*X*^2^ value	*p* value
Age	< 56	8	4	(50.0)	4	(50.0)	1.49	0.222
	≥ 56	46	33	(71.7)	13	(28.3)		
Gender	Male	40	27	(67.5)	13	(32.5)	0.07	0.785
	Female	14	10	(71.4)	4	(28.6)		
Differentiation	Well	15	15	(100.0)	0	(0.0)	13.42	0.001**
	Moderate	18	13	(72.2)	5	(27.8)		
	Poor	21	9	(42.9)	12	(57.1)		
T stage	T1 T	24	24	(100)	0	(0.0)	15.83	0.001**
	T2	7	3	(42.9)	4	(57.1)		
	T3	15	8	(53.3)	7	(46.7)		
	T4	6	4	(66.7)	2	(33.3)		
N stage	N0	37	30	(81.1)	7	(18.9)	12.20	0.007**
	N1 N2	6	3	(50.0)	3	(50.0)		
	N2	3	0	(0.0)	3	(100.0)		
	N3	7	3	(42.9)	4	(57.1)		
M stage	M0	54	37	(68.5)	17	(31.5)	-	-
	M1	0	0	(-)	0	(-)		

The association between MCAM expression and various clinicopathological features in patients with GC. Statistical comparisons were performed as follows: (1) Differentiation, MCAM expression was compared across well, moderately, and poorly differentiated tumors. The result (*p* = 0.001) indicates that MCAM expression is significantly higher in poorly differentiated tumors than in well and moderately differentiated ones. (2) T stage (tumor invasion depth), we compared MCAM expression between early-stage (T1–T2) and advanced-stage (T3–T4) tumors. A higher MCAM expression was observed in T3–T4 tumors, with a significant difference (*p* = 0.001) compared to T1–T2. (3) N stage (lymph node involvement), MCAM expression was analyzed among N0–N1 (less/mild lymph node involvement) and N2–N3 (extensive lymph node involvement) cases. The data (*p* = 0.007) show that MCAM levels are significantly higher in N2–N3 cases than in N0–N1 cases. For age and gender, no significant associations with MCAM expression were found (*p* > 0.05). The χ^2^ test was used for these statistical analyses, and “**” denotes statistical significance.

### Overexpression of MCAM significantly promotes GC cell proliferation and migration

MCAM may play a crucial role in the aggressive behavior of GC cells.

**Figure 2.**
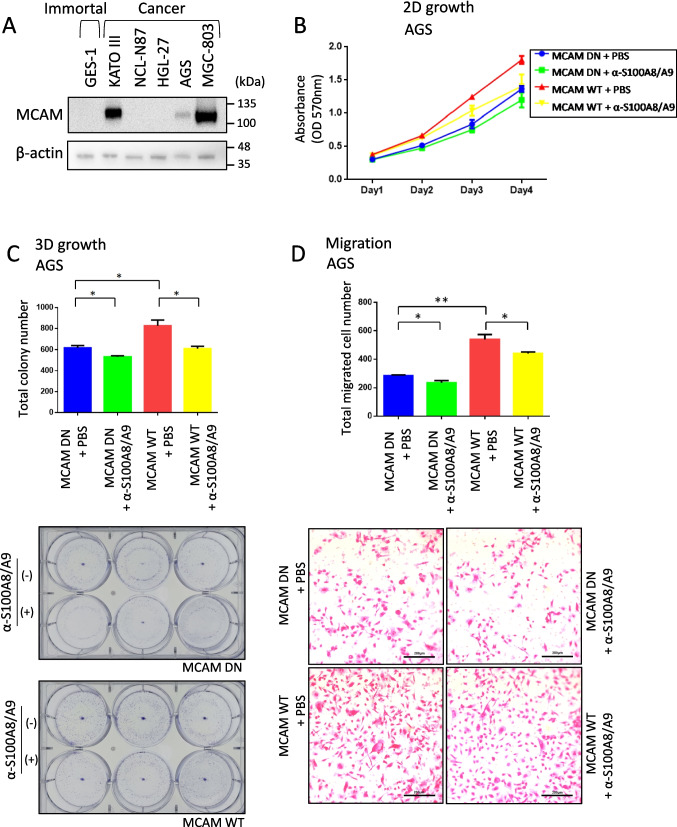
Extracellular S100A8/A9 promotes GC cell growth and migration via MCAM. (*A*): Western blot analysis of MCAM expression in GC cell lines. MCAM protein was detected in protein extracts from various gastric cell lines. GES-1 is a human immortalized non-cancerous gastric epithelial cell line; KATO III, NCL-N87, HGL-27, AGS, and MGC-803 are GC cell lines. (*B*, *C*, *D*:) In vitro cell-based assays using AGS cells, which exhibit moderate MCAM expression. AGS cells transfected with either MCAM wild -type (MCAM WT) or dominant-negative MCAM (MCAM DN) 35 constructs were evaluated for standard 2D growth using an MTT assay over time, in the presence or absence of S100A8/A9 antibody (1 μg/ml) under co-treatment with recombinant S100A8/A9 (0.1 μg/ml) in the culture medium. Colony formation (3D growth) was assessed similarly to (*B*), with quantification shown in bar graphs (*top*) and representative images of colony formation (*bottom*). Cell migration was evaluated using a Boyden chamber assay (*D*), with 0.1 μg/ml S100A8/A9 placed in the lower chamber with or without 1 μg/ml S100A8/A9 antibody. Quantified data and representative images of migrated cells are shown. Data are presented as mean ± SD. ND,: not detected. *p<0.05, **p<0.01 by Student’s *t*-test.

We systematically investigated the functional role of MCAM in GC. We first analyzed its expression in a panel of GC cell lines using WB (Fig. 2*A*). MCAM was barely detectable in the non-cancerous immortalized gastric epithelial cell line GES-1 but was readily expressed in KATO III, AGS, and MGC-803 GC cell lines. Among the three MCAM-positive cell lines, MGC-803 cells showed the highest endogenous expression of MCAM, whereas AGS cells exhibited the lowest level based on band intensity. Therefore, AGS cells were selected for MCAM overexpression studies, and conversely, MGC-803 cells were used for MCAM knockdown experiments.

We next evaluated the effects of MCAM expression on anchorage-dependent GC cell growth. We assessed the effects of MCAM on GC cell anchorage-dependent growth activity under the standard cultivation method (two-dimensional (2D)-growth) using an MTT assay (Fig. 2*B*). In AGS cells, overexpression of wild-type MCAM (MCAM WT) significantly enhanced cell growth at days 2, 3, and 4 compared to dominant-negative MCAM (MCAM DN) (*p* < 0.001 ***) in the presence of recombinant S100A8/A9 in the culture medium. Interestingly, treatment with an anti-S100A8/A9 neutralizing antibody (α-S100A8/A9) suppressed cell growth in both MCAM WT and MCAM DN groups at days 3 and 4. Specifically, cell growth was significantly inhibited in the MCAM DN + α-S100A8/A9 group compared to MCAM DN alone (*p* = 0.032 *) and in the MCAM WT + α-S100A8/A9 group compared to MCAM WT alone (*p* = 0.0053*), suggesting that S100A8/A9 promotes proliferation via MCAM downstream signaling upon S100A8/A9 stimulation. Because anchorage-independent growth (3D-growth) is a more inherent trait of cancer cells than increased activity in 2D growth, a colony formation assay was conducted for the MCAM-overexpressing cells (Fig. 2*C*). AGS cells overexpressing MCAM formed significantly more and larger colonies in both number and size than those of the MCAM DN group. This enhanced colony formation was attenuated by S100A8/A9 antibody treatment in both the MCAM WT and MCAM DN groups, further supporting the involvement of S100A8/A9 in MCAM-mediated tumor-promoting effects.

Analysis of migrative capacity provides additional insight into GC cell aggressiveness. We then performed transwell migration assays (Fig. 2*D*). Overexpression of MCAM in AGS cells markedly increased migratory ability compared to the MCAM DN group. However, treatment with the anti-S100A8/A9 antibody significantly reduced migratory capacity in both the MCAM WT and MCAM DN groups, indicating that S100A8/A9 mediates, at least in part, the MCAM-driven migratory phenotype in GC cells. These results demonstrate that MCAM promotes GC cell growth and migration and that these effects are, at least partially, mediated through S100A8/A9 signaling.

### MCAM knockdown suppresses GC cell proliferation and migration

**Figure 3.**
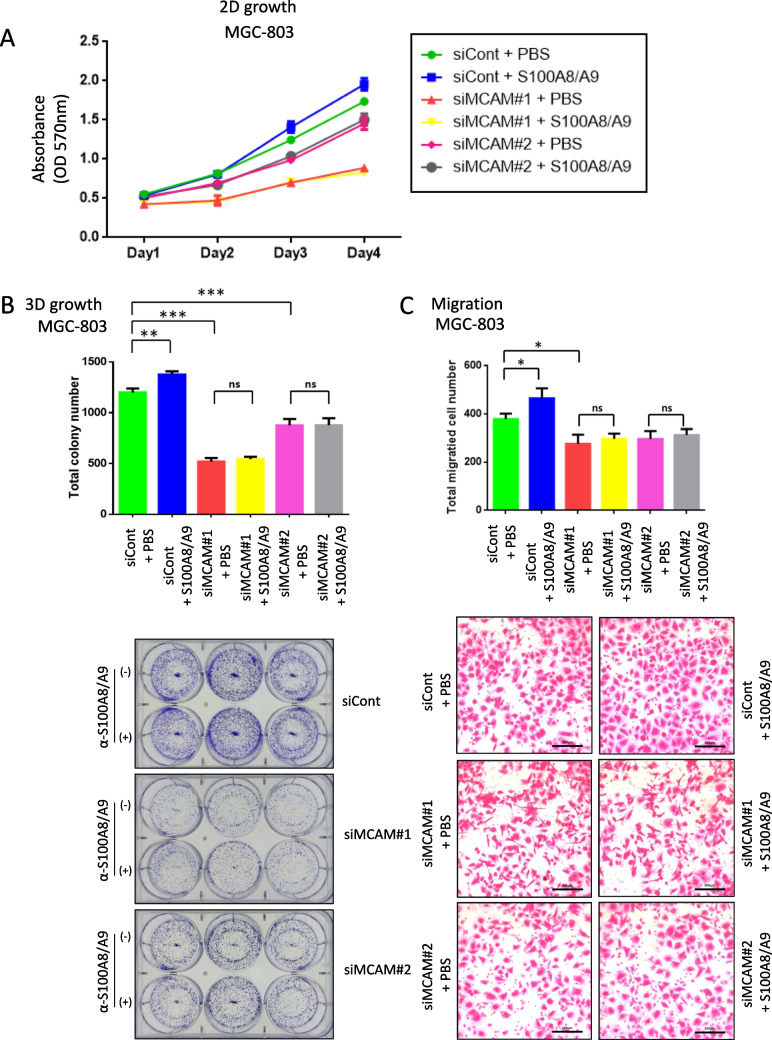
Knockdown of MCAM suppresses GC cell growth and migration. (*A*, *B*, *C*:) In vitro cell-based assays using MGC-803, which exhibited the highest MCAM expression among the GC cell lines employed (Figure 2*AA*). (*A*:) MTT assay to evaluate the 2D growth of MGC-803 cells over time. Cells were transfected with the indicated siRNAs (siCont, siMCAM #1, or siMCAM #2) and continuously cultured with or without S100A8/A9 (0.1 μg/ml) in the culture medium. (*B*): Colony formation assay to evaluate 3D growth. siRNA-treated cells, prepared as described in (*A*), were cultured under non-adherent conditions. The formed spheroids were quantified based on diameter (*B*). Quantified *bar graphs* and representative images of colony growth are shown in the *top* and *bottom panels*, respectively. (*C*): 36 Migration assay using the Boyden chamber method. MGC-803 cells transfected with the indicated siRNAs were assessed for migration with or without S100A8/A9 (0.1 μg/ml) in the lower chamber. Quantified results are shown in the *upper panel*, and representative images of migrated cells are shown in the *bottom panel*. Data are presented as mean ± SD. ND,: not detected. *p < 0.05, * *p < 0.01, * * *p < 0.001 by Student's’s *t*-test.

Our loss-of-function experiments using siRNA-mediated knockdown in MGC-803 cells, which express abundant MCAM at the protein level, revealed the functional role of MCAM in GC cells. As shown in (Fig. 3*A*), treatment with recombinant S100A8/A9 protein significantly promoted cell growth on days 3 and 4 in the siCont group (*p* = 0.0015**). However, MCAM knockdown by two independent siRNAs (siMCAM#1 and siMCAM#2) significantly suppressed cell growth relative to the control (*p* < 0.001*** for both). Importantly, the addition of S100A8/A9 did not restore growth in MCAM-deficient cells, as no significant difference was observed between siMCAM#1 and siMCAM#1 + S100A8/A9 (*p* = 0.1194) or between siMCAM#2 and siMCAM#2 + S100A8/A9 (*p* = 0.3691). Similarly, colony formation assays (Fig. 3*B*) showed that MCAM knockdown significantly decreased both the number and size of colonies. Again, S100A8/A9 stimulation did not reverse this effect, indicating that MCAM is essential for tumor-promoting responses to extracellular S100A8/A9 in GC cells. These findings highlight the essential role of MCAM in mediating S100A8/A9-driven growth responses. In agreement with these results, transwell assays (Fig. 3*C*) demonstrated that MCAM knockdown significantly impairs cell migration. Furthermore, supplementation with S100A8/A9 failed to restore migratory capacity in MCAM-silenced cells, suggesting that MCAM is required for S100A8/A9-mediated chemotactic motility.

### MCAM triggers ERK/c-Jun pathway activation upon S100A8/A9 binding

**Figure 4.**
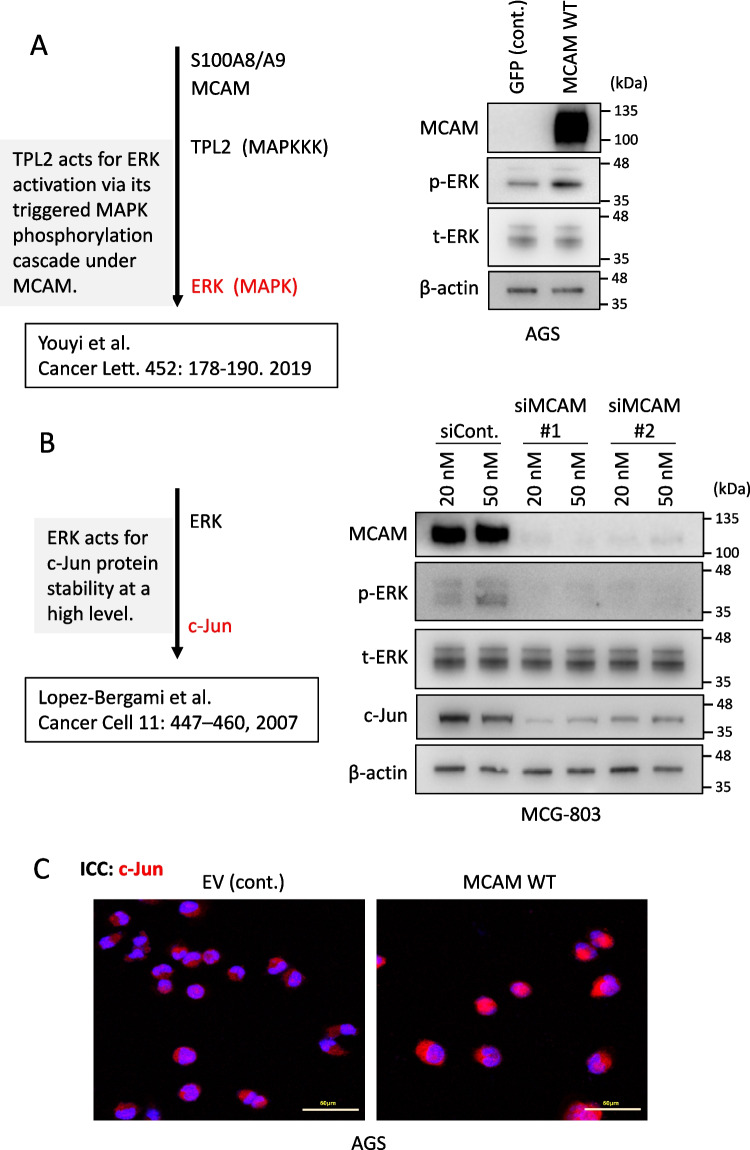
MCAM induces ERK/c-Jun pathway upon S100A8/A9 binding. ( *A*, *B*:) Western blot analysis of MCAM downstream signaling in GC cell lines AGS (*A*) and MGC-803 (*B*). A simplified diagram of the MCAM-mediated oncogenic signaling pathway is shown (*A*, *left*). AGS cells were transiently transfected with MCAM WT or control GFP and treated with S100A8/A9 (0.1 μg/ml) (*A*, *right*). A schematic of the canonical ERK–c-Jun pathway is shown (*B*, *left*). MGC-803 cells were transfected with the indicated siRNAs at different concentrations and stimulated with S100A8/A9 (0.1 μg/ml) (*B*, *right*). (C: ) Immunocytochemistry of c-Jun in AGS cells. Cells prepared under the same conditions as in (*A*), except using an empty vector (EV) instead of GFP as a control, were immunofluorescence-stained with anti–c-Jun antibody (*red*). Nuclei were counterstained with DAPI (*blue*). *Scale bars*: 50 μm.

**Figure 5.**
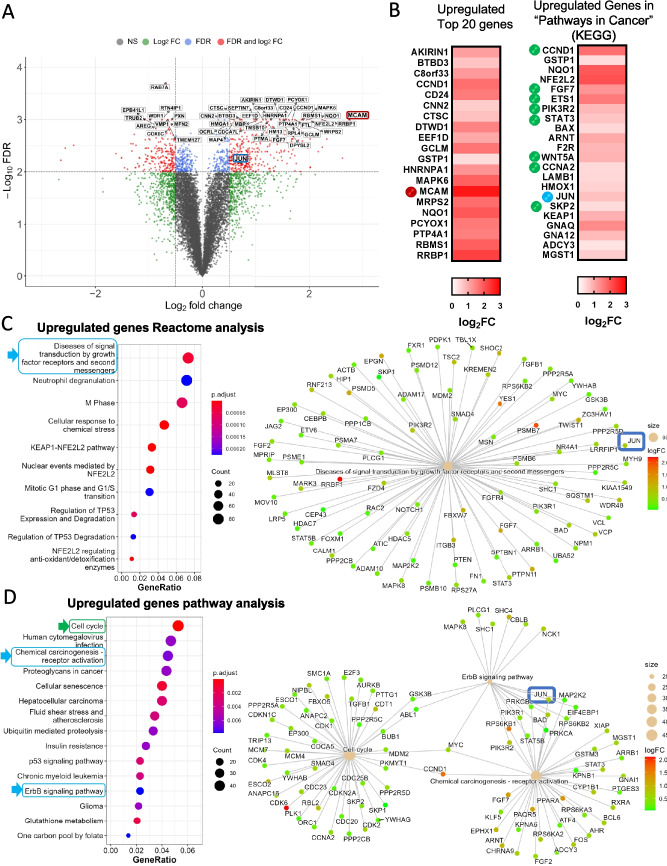
Comprehensive analysis of gene expression alterations induced byextracellular S100A8/A9 in GC cells. (*A*-*D*): RNA-Seq analysis. RNA-Seq was performed on MGC-803 cells treated (*n*=3) or untreated (*n*=3) with S100A8/A9 (0.1 μg/ml). (*A*): Following the analysis, a volcano plot was generated to visualize significantly altered gene expression. MCAM and JUN (c-Jun) are *encircled in red and blue*, respectively. (*B*): Heat maps of upregulated genes in S100A8/A9-treated MGC-803 cells. The *left panel* displays the top 20 upregulated genes. The *right panel* shows genes upregulated in the KEGG-defined category ‘pathways in cancer’. MCAM is highlighted with a *red circle*, JUN with a *blue circle*, and additional cancer-relevant genes of interest are circled in *green*. These analyses were conducted using the Database for Annotation, Visualization, and Integrated Discovery (DAVID) v6.8 (http://david.ncifcr.gov/). All statistical analyses were conducted using R (ver. 4.1.0). (*C*, *D*:) In silico pathway analyses using Reactome (*C*) and general pathway enrichment (*D*). The upregulated genes classified under the same functional categories in ‘Reactome analysis’ (*C*) and ‘Pathway analysis’ (*D*) converge on various cancer-relevant processes, marked with *blue arrows* and *circles* in the *left panels*, including the JUN (c-Jun) gene, which is encircled by *blue* in the *right panels*. Notably, the gene set with the highest number of associated genes was linked to the ‘cell cycle’ pathway, which ranked at the top (*D*).

Our investigation into the molecular mechanism of the S100A8/A9–MCAM-mediated downstream signaling pathway, which promotes GC cell growth and migration, was conducted with a clear and coherent experimental strategy. We particularly focused on ERK, as its activation is positively regulated by TPL2 under MCAM following binding to S100A8/A9 (Chen* et al.* 2019b). The WB result (Fig. 4*A*) in AGS cells, where ectopic overexpression of the MCAM wild-type plasmid led to robust MCAM expression and a concomitant increase in ERK phosphorylation in the presence of recombinant S100A8/A9 in the culture medium, provided clear evidence. ERK activation enhances the protein stability of the oncogenic transcription factor (TF) c-Jun, a component of this signaling cascade (Lopez-Bergami* et al.* 2007). MCAM knockdown in MGC-803 cells using two independent siRNAs (siMCAM#1 and siMCAM#2) significantly and consistently reduced MCAM protein expression, accompanied by a marked decrease in phosphorylated ERK (p-ERK) levels even in the presence of S100A8/A9 (Fig. 4*B*). In this case, we confirmed that phosphorylation levels of another effector kinase, p38 MAPK, remained unchanged, while JNK phosphorylation, which contributes to c-Jun activation, was observed (data not shown). Along with the ERK activation fluctuation, the expression of c-Jun also fluctuated accordingly. In line with this, immunofluorescence staining demonstrated enhanced nuclear localization of c-Jun in AGS cells overexpressing MCAM in response to S100A8/A9, confirming its activation at the protein level (Fig. 4*C*). These findings support a S100A8/A9‒MCAM-ERK‒c-Jun pathway that operates in GC cells (Fig. 5).

### S100A8/A9 regulates multiple oncogenic genes in MCAM-positive GC cells

ERK targets multiple cancer-relevant proteins other than c-Jun. Similarly, c-Jun regulates the expression of multiple oncogenic genes, which may mutually orchestrate one another as a tumor-promoting cascade in GC progression. We investigated changes in gene expression after stimulation of MGC-803 cells with S100A8/A9 in a comprehensive manner using the RNA-Seq method. This approach yielded insights into S100A8/A9-regulated gene expression profiles relevant to cancer progression. We first observed that c-Jun is induced by S100A8/A9 at the mRNA level, as shown by volcano scatter plots (Fig. 5*A*, encircled in blue).

**Figure 6.**
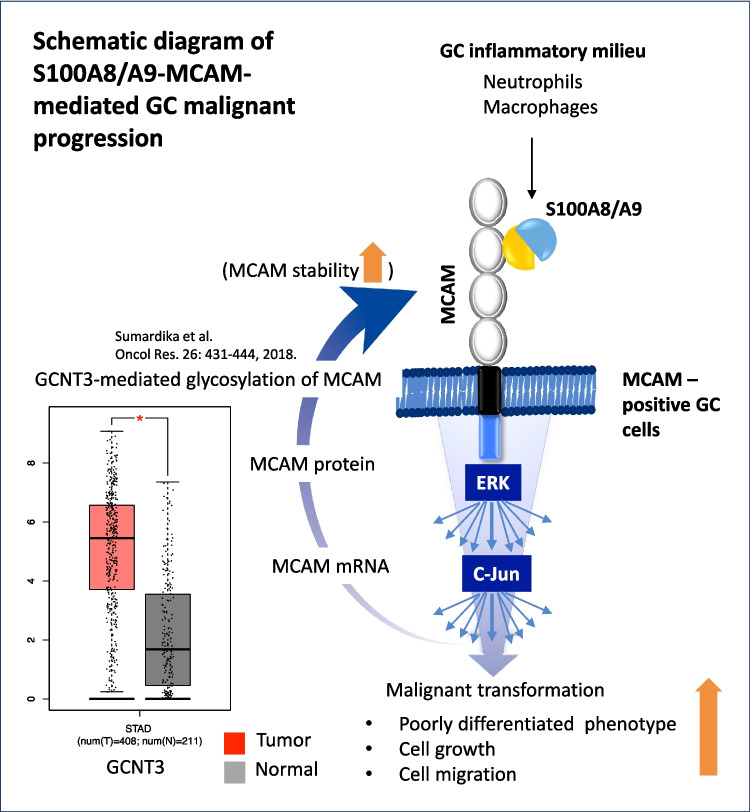
Schematic abstract of the S100A8/A9‒MCAM-mediated GC progression. As the GC milieu is inherently inflammatory, the active secretion of S100A8/A9 by infiltrated neutrophils and macrophages becomes a key factor in fueling GC cells via the S100A8/A9 receptor MCAM on MCAM-positive cells. The binding of S100A8/A9 to MCAM triggers an oncogenic ERK–c-Jun signaling cascade and potentially other pathways, which act in concert to contribute to the activation of multiple oncogenic genes. This collaborative nature of the pathways involved in GC progression underscores the complexity and interconnectedness of our research. Notably, MCAM mRNA is re-expressed by the revealed pathway at a significant level, and the GCNT3-mediated glycosylation process may further stabilize the translated MCAM protein, resulting in a high level of MCAM protein on the surface of GC cells. The feed-forward loop of the S100A8/A9–MCAM–ERK-c-Jun signaling pathway emerges as a key regulator of malignant progression in GC.

Regarding this observation, the same plot showed MCAM induction at a significant level, as confirmed by its appearance in the heat map image composed of the top 20 upregulated genes Fig. 5*B*, left panel, marked by a red circle). The S100A8/A9-upregulated gene set matched to “pathways in cancer” in the pathway analysis using the KEGG database and included c-Jun (blue circle) and a series of well-established cancer-relevant genes (green circle), such as cell cycle enhancers (CCND1 (cyclin D1), CCNA2 (cyclin A2), and SKP2), FGF signaling (FGF7), transcription factors (ETS1 and STAT3), and Wnt–beta-catenin signaling (WNT5A) (Fig. 5*B*, right panel). Moreover, when focusing on cancer-relevant pathways that include c-Jun, we found that three interesting pathways closely involved in cancer progression were highlighted (Fig. 5*C* and *D*), blue arrows and circles). In addition, as shown in (Fig. 5*D*), the pathway “cell cycle” was placed at the very top, indicating that the largest number of S100A8/A9-upregulated genes mapped to the “cell cycle” category (green arrows and circles), which is essential to the acceleration of cancer growth. Together, these results support the idea that MCAM contributes to the malignant transformation of GC cells via positive regulation of cell growth and migration upon S100A8/A9 binding, which requires the ERK–c-Jun pathway at least in part. The signaling pathway promotes the expression of multiple oncogenic genes, several of which may also enhance MCAM expression, forming a self-reinforcing feed-forward loop. This sustained activation likely plays a central role in driving GC progression (Sumardika* et al.* 2018) (Fig. 6).

## Discussion

GC continues to pose a formidable global health challenge, characterized by its high incidence and mortality rates. The intricate interplay of numerous molecular and cellular mechanisms underlies the development and progression of GC, making it a complex disease to understand and treat (Siegel* et al.* 2022). In recent years, an increasing body of research, including our collaborative efforts, has focused on the roles of S100A8 and S100A9 proteins in GC, shedding light on their potential as critical contributors to tumorigenesis. The present study demonstrates that the heterodimer complex of S100A8 and S100A9 (S100A8/A9) plays a crucial role in GC progression through MCAM binding, which activates downstream ERK‒c-Jun signaling and induces the upregulation of several oncogenic genes that likely interact in promoting the malignant phenotype. Re-induction of MCAM at elevated levels appears to be a critical event in malignant GC progression, as it establishes a feed-forward loop that amplifies MCAM-mediated signaling in a self-reinforcing manner.

First, we discuss S100A8/A9, which has emerged as a critical factor in various cancers, including GC (Bresnick *et al*. 2015). These proteins typically form a heterodimer, calprotectin, which is more abundant in vivo than the individual S100A8 and S100A9 monomers or their homodimers (Tardif *et al*. 2015). The primary cellular sources of its production and secretion in the cancer inflammatory milieu are neutrophils (Wu *et al*. 2020; Xiong *et al*. 2021). Besides neutrophils, monocytes, macrophages, and myeloid-derived suppressor cells (MDSC) are also important contributors to S100A8/A9 production (Wang *et al*. 2013; Zheng *et al*. 2015). Compiling evidence indicates that S100A8/A9 levels are significantly elevated in tumor lesions compared to adjacent normal gastric mucosa, with strong infiltration of S100A8/A9-producing cells within tumor regions (Fang *et al*. 2024), which increases in proportion with tumor grade (Zhang *et al*. 2023). This trend is also evident in GC, where S100A8/A9 levels rise with disease stage, particularly in poorly differentiated tumors, which show markedly increased MCAM expression in our study (Fig. 1*D*). The elevated levels of the ligand S100A8/A9 and its receptor MCAM likely contribute to the development of aggressive, poorly differentiated GC, which is associated with reduced patient survival (Fig. 1*E*). The extracellular S100A8/A9 fosters tumor growth, angiogenesis, and metastasis (Srikrishna 2012). In addition, abundant extracellular S100A8/A9 can recruit more S100A8/A9-producing cells into the tumor site, where they continue to secrete S100A8/A9 along with inflammatory cytokines, chemokines, and growth factors, thus forming a sustained positive feed-forward loop that efficiently promotes cancer cell proliferation and invasion. This mechanism is relevant to GC, as S100A8/A9 has been shown to induce the epithelial–mesenchymal transition (EMT) in GC cells, enhancing their migratory and invasive capabilities—effects in which S100A8/A9–MCAM signaling plays a key role.

Our second focus is the S100A8/A9 receptor MCAM, a cell surface glycoprotein with diverse roles in physiological and pathological processes (Wang *et al*. 2020; Miller *et al*. 2024; Braun *et al*. 2025). In GC, MCAM expression is upregulated, and its levels are positively correlated with tumor aggressiveness, lymph node metastasis, and overall poor patient outcomes. The interaction between S100A8/A9 and MCAM represents a crucial molecular event in GC progression. Our RNA-Seq approach revealed an interesting result that S100A8/A9 induces MCAM expression at significant levels (Fig. 5*A* and *B*), which are reflected at the protein level in GC cells (Fig. 1*C* and *D*, Fig. 2*A*). The translated MCAM protein likely becomes highly stable through glycosylation mediated by glucosaminyl (N-acetyl) transferase 3, mucin type (GCNT3), which we previously reported in melanoma (Sumardika *et al*. 2018); notably, GCNT3 is also highly expressed in GC (Fig. 6). GCNT3-stabilized MCAM in GC cells may functionally differ from the baseline MCAM expressed in interstitial stromal cells of normal gastric mucosa (Fig. 1*A*) due to markedly higher protein levels that enhance S100A8/A9 TV signal strength and stream oncogenic effects (Wang *et al*. 2020). Hence, MCAM is conceivably capable of amplifying S100A8/A9 signals to promote GC progression when MCAM is strongly upregulated in GC cells.

The third critical point is the presence of an oncogenic ERK–c-Jun pathway regulated by the S100A8/A9–MCAM axis. The ERK–c-Jun signaling axis has long been recognized as a central regulator of cell growth, survival, and differentiation (Lopez-Bergami *et al*. 2007). In the context of GC, its dysregulated hyperactivation is closely associated with tumorigenesis and disease progression. While this pathway can be activated by multiple oncogenic tyrosine kinase receptors, including EGFR and ERBB2 (Baselga *et al*. 2009), our siRNA-mediated knockdown approach targeting MCAM notably diminished both ERK activation and c-Jun induction substantially, suggesting that the S100A8/A9–MCAM axis strongly regulates this pathway in GC cells. This raised the question of how ERK activation leads to c-Jun upregulation. As shown in the left panel of Fig. 4*B*, Lopez-Bergami *et al*. (2007) reported that c-Jun induction and stability are positively regulated by activation of the CREB transcription factor (which drives c-Jun at mRNA expression) and GSK inactivation (which stabilizes c-Jun protein), both mediated by ERK-dependent phosphorylation. These findings align with our results, in which c-Jun is upregulated at both mRNA (Fig. 5*A* and *B*) and protein (Fig. 4*B* and *C*) levels following MCAM activation in response to S100A8/A9 binding. The increased level of c-Jun is essential for cell cycle promotion through the induction of cyclin D1 (Lopez-Bergami *et al*. 2007). Our RNA-Seq result revealed that CCND1 (cyclin D1) is upregulated at a significant level (Fig. 5*B*), ranking the “cell cycle” progression pathway at the highest level (Fig. 5*D*). Although our RNA-Seq analysis did not highlight a metastasis-relevant pathway, ERK–c-Jun activation can induce the expression of matrix metalloproteinases (MMPs), which degrade the extracellular matrix and facilitate tumor cell invasion and metastasis (Visse *et al*. 2003).

We therefore propose that targeting the S100A8/A9‒MCAM‒ERK-c-Jun axis holds strong potential as a therapeutic strategy for GC. In support of this, our S100A8/A9-neutralizing antibody, Ab45 (Kinoshita *et al*. 2019), has demonstrated efficacy in reducing proliferation and migration of MCAM-positive GC cells in vitro (Fig. 2*B*, *C*, *D*, and Fig. 3). These findings suggest that Ab45 may serve as a promising therapeutic tool for treating GC in the future.

## Conclusion

The S100A8/A9‒MCAM‒ERK-c-Jun signaling axis plays a critical role in GC progression by establishing a positive feedback loop via MCAM re-expression, which promotes GC cell proliferation and invasion. Disrupting this loop through targeted therapies may represent a promising approach to improving GC treatment outcomes. Further research is needed to evaluate the clinical applicability of these findings and to advance therapeutic strategies targeting this pathway. Our S100A8/A9-neutralizing antibody, Ab45 (Kinoshita *et al*. 2019), is a promising candidate that supports the translational potential of this research.

## Data Availability

The raw data supporting the conclusions of this article will be made available by the authors, without undue reservation.
